# Connecting the Dots: Health Information Technology Expansion and Health Disparities

**DOI:** 10.1371/journal.pmed.1001852

**Published:** 2015-07-14

**Authors:** Courtney Lyles, Dean Schillinger, Urmimala Sarkar

**Affiliations:** 1 Division of General Internal Medicine, San Francisco General Hospital, Department of Medicine, University of California San Francisco, San Francisco, California, United States of America; 2 Center for Vulnerable Populations, Department of Medicine, San Francisco General Hospital, University of California San Francisco, San Francisco, California, United States of America

## Abstract

Courtney Lyles and colleagues highlight how the expansion of patient-facing electronic health record portals could exacerbate existing healthcare disparities.

**Summary Points**

- Meaningful use is an incentive program sponsored by the US federal government that has provided more than US$25 billion to date to incentivize US healthcare clinics and hospitals to implement electronic health records (EHRs). Healthcare systems receive incentives for reaching a wide range of EHR targets, including providing patient access to/use of EHR information through portal websites.
- Early evidence links EHR and portal use to better healthcare processes and health outcomes.
- Promoting patient engagement with health technology such as portals is challenging, and rapid expansion of portals could exacerbate existing healthcare disparities if only well-resourced individuals use these websites.
- Improving the usability and accessibility of portals for diverse patients requires collaboration between health communication researchers, user-centered designers, healthcare systems, vendors, and government agencies.

Both in the United States and internationally, there is a huge push to implement integrated electronic health records (EHRs). This adoption of health technology is viewed as critical to improving healthcare quality, and studies have shown that EHR implementation is linked to higher receipt of appropriate processes of care [1,2]. In the US, federal healthcare reform legislation jumpstarted this transition to EHRs, largely because providers in the fragmented healthcare marketplace did not have aligned financial incentives to modernize their medical records on their own. The Health Information Technology for Economic and Clinical Health (HITECH) Act in 2009 created the multi-billion-dollar EHR Incentive Program, the meaningful use program, which is managed by the Office of the National Coordinator for Health Information Technology (ONC). The meaningful use program has been very successful, with 94% of US hospitals receiving payment for EHR implementation [3] and 77% of US office-based health professionals receiving payment [4].

However, while primarily intended for system and provider implementation of EHRs, the meaningful use program also includes targeted metrics for patient-level engagement and use of electronic medical data. Beginning in 2014, US healthcare clinics and systems will receive federal incentives for the following: having 50% of their eligible patient population registered for access to a patient-facing portal website linked to the EHR; having 5% of their eligible patient population actively viewing, downloading, and transmitting health information through this portal website; and providing patient educational materials on these websites [5]. Despite the large amount of money on the table, almost all healthcare systems are struggling with patient portal use, so much so that the meaningful use program granted a one-year extension through the end of 2015 for systems to meet early patient engagement goals [6]. These current challenges with patient engagement raise two important questions about widespread health technology such as EHRs: Are we designing EHRs/portals that patients can and want to access? And are specific groups, such as those with limited literacy or other communication barriers (limited health literacy or English proficiency), facing even greater barriers to portal use? The clinical vignette provided in Box 1 illustrates some of the challenges with the current implementation approach.

Health technologies like portals have great potential to improve healthcare quality and efficiency by enhancing communication between patients/caregivers and healthcare providers. Early evidence links patient portal use to improved health outcomes such as better diabetes control and medication adherence [7–9]. Specifically, portals can lower barriers to engaging in health-related tasks by increasing convenience and access to medical record information and tools online (e.g., reviewing test results online at any time rather than calling a provider/clinic for information). This improved communication and care coordination is particularly important for patients with chronic illness because they need increased assistance managing complex self-management tasks, and chronic illness disproportionately affects more vulnerable patient subgroups. Thus, many have argued that patient use of health technology such as portals could reduce health disparities related to race/ethnicity and limited health literacy [10].

**Box 1. Clinical Vignette**

Maria is a 50-year-old, primarily Spanish-speaking patient with diabetes seen in a safety net healthcare system in California. At her latest visit with her primary care doctor (conducted in Spanish), her doctor adds insulin to her treatment regimen. He talks a lot about the new medication, tells her he is scheduling an appointment with a diabetes educator, and hands her several pages of information, in Spanish, about starting insulin. When she leaves the office, he mentions a visit summary available through the clinic’s new portal website. However, when she gets home and tries to sign up, she is unable to carry out the registration process on the English-only website. When her son finally helps her log on, she realizes that the visit summary section contains very general information about diabetes, but none of the specific instructions her doctor gave her during the visit—and she is now struggling to remember the details of their conversation. She tells her son that during the visit with her doctor, she felt scared and overwhelmed about starting insulin, and it made it hard to focus on what the doctor said.

However, portal expansion is not yet fully realizing this promise. Racial/ethnic minority groups and those with limited literacy have consistently been shown to be less likely to use Internet-based patient portals in healthcare systems that were early adopters of this technology [11,12]. Evidence suggests that this is not an issue of access or interest alone: almost all Americans have Internet access (across demographic subgroups such as income and race/ethnicity), and the vast majority of patients across healthcare settings are interested in Internet-based communication with providers or health systems [13].

We believe lack of usability is a formidable barrier to achieving widespread use of portals and other patient-facing health technology, particularly for diverse groups. Although usability data for portals remain relatively sparse, the few formal studies that have been conducted demonstrate that portals are difficult to use, with multiple challenges involved in apparently straightforward tasks, such as requesting access to the site, and in more complex tasks, such as comprehension of the medical information presented [14,15]. These challenges are amplified among some vulnerable patient populations. In a recent study of a racially/ethnically diverse group of 51 older adults, 86% of participants shown a video documenting the available features of a portal website stated that they would use it, but only 12% were able to correctly complete a set of simple tasks during a simulation, and none were able to complete a set of complex tasks [16].

Similarly, portals can amplify the existing challenges of patient—provider communication during and between visits. In 2011 the Institute of Medicine commissioned an important paper stating that healthcare organizations have a responsibility for reducing the complexity of the healthcare system and defining the attributes of health-literate healthcare systems [17]. One of the attributes was designing and distributing content that was easy to understand and act on. However, text—such as the patient education materials provided within portals—is rarely written at a lower literacy level or available in a wide range of languages. Two required portal features in the meaningful use criteria—visit summaries and lab results—also often contain confusing technical language [18,19]. Healthcare providers often find themselves in the uncomfortable position of needing to comply with the meaningful use mandate and therefore delivering EHR-generated visit summaries that are full of medical jargon, do not reinforce their recommendations, and do not enhance comprehension.

Moving forward, those of us in the medical community can advocate for many improvements in the usability of portals to ensure they are relevant to diverse groups. For example, when we design or implement health technologies, we can apply principles from product design and health communication science. User-centered product design involves understanding the needs, values, and abilities of users to improve the quality of users’ interactions with and perceptions of the technology. This strategy has been largely nonexistent when developing or testing portals. In addition, communication researchers have long demonstrated that online platforms have the potential to address disparities in language and health literacy, largely because they can leverage audio and video to enhance engagement, can more seamlessly provide non-English language access, and can provide interactivity and feedback to optimize comprehension.

There is also an opportunity for federal agencies such as the ONC (who oversees the meaningful use program) to address the usability and accessibility of portals. Importantly, this agency has included patient engagement within the meaningful use metrics and has laid out several goals for increasing patient understanding and empowerment, including in its draft *Federal Health IT Strategic Plan 2015–2020* [20,21]. We share this vision, but contend that these goals can be reached only by designing, testing, and evaluating technology specifically for and with diverse populations. We believe that the ONC could make its goals more actionable by funding or supporting usability testing among diverse populations with significant health needs, by creating meaningful use standards for literacy and language appropriateness for patient information such as visit summaries, and by incentivizing broad implementation of portal interfaces in multiple languages.

Finally, we see an opportunity for the marketplace to capitalize on these challenges with long-term patient engagement to differentiate products based on how well they are able to improve understanding and decision-making among patients and providers. Healthcare providers and hospital systems, as the “consumers” of EHR products, should look to purchase EHRs with patient-facing portal products that meet the basic needs of their patient population; language and literacy should not be considered “add-on” or “extra” features for already costly EHRs. Similarly, EHR/portal programmers and developers should familiarize themselves with the issues of health literacy and healthcare disparities, perhaps by establishing and consulting with patient advisory groups that include bilingual patients and individuals across the socioeconomic status spectrum. Ensuring that a portal is accessible for those with limited literacy or language barriers can improve the product overall, and should be seen as a starting place for innovation and competitive advantage for EHR vendors.

The timeliness of rapid EHR implementation makes this a critical juncture for technology and disparities in policy as well as clinical practice. In addition to being vigilant about fixing the technical glitches and basic operability of EHRs, we must also be deliberate about addressing the much harder task of patient engagement with portals and other health technologies. We call on policymakers, healthcare leaders, and the private sector to engage with the experts in the health disparities and communication science fields to start to bridge the digital divide now. The US example of portal use will likely become an illustrative example of many issues that will be faced in the dissemination of patient technologies across countries or settings serving a diverse patient population.

## Abbreviations

EHR: electronic health record
ONC: Office of the National Coordinator for Health Information Technology
